# Alkylbenzoic and Alkyloxybenzoic Acid Blending for Expanding the Liquid Crystalline State and Improving Its Rheology

**DOI:** 10.3390/ijms242115706

**Published:** 2023-10-28

**Authors:** Anastasiya Y. Yadykova, Ivan I. Konstantinov, Anna V. Vlasova, Lydia A. Varfolomeeva, Sergey O. Ilyin

**Affiliations:** A.V. Topchiev Institute of Petrochemical Synthesis, Russian Academy of Sciences, 29 Leninsky Prospect, 119991 Moscow, Russia

**Keywords:** liquid crystals, mesophases, nematics, smectics, *p*-alkylbenzoic acids, *p*-alkyloxybenzoic acids, noncovalent dimers, heterodimers, viscosity

## Abstract

Thermotropic mesogens typically exist as liquid crystals (LCs) in a narrow region of high temperatures, making lowering their melting point with the temperature expansion of the mesophase state an urgent task. *Para*-substituted benzoic acids can form LCs through noncovalent dimerization into homodimers via hydrogen bonds, whose strength and, consequently, the temperature region of the mesophase state can be potentially altered by creating asymmetric heterodimers from different acids. This work investigates equimolar blends of *p*-*n*-alkylbenzoic (*k*BA, where *k* is the number of carbon atoms in the alkyl radical) and *p*-*n*-alkyloxybenzoic (*k*OBA) acids by calorimetry and viscometry to establish their phase transitions and regions of mesophase existence. Non-symmetric dimerization of acids leads to the extension of the nematic state region towards low temperatures and the appearance of new monotropic and enantiotropic phase transitions in several cases. Moreover, the crystal–nematic and nematic–isotropic phase changes have a two-step character for some acid blends, suggesting the formation of symmetric and asymmetric associates from heterodimers. The mixing of 6BA and 8OBA most strongly extends the region of the nematic state towards low temperatures (from 95–114 °C and 108–147 °C for initial homodimers, respectively, to 57–133 °C for the resulting heterodimer), whereas the combination of 4OBA and 5OBA gives the most extended high-temperature nematic phase (up to 156 °C) and that of 6BA and 9OBA (or 12OBA) provides the existence of a smectic phase at the lowest temperatures (down to 51 °C).

## 1. Introduction

Liquid crystals (LCs) or mesomorphic systems are of great interest to fundamental science and applied industrial technologies because of their unique properties. Like isotropic liquids (I), LCs have fluidity and the ability to be in a droplet state but, at the same time, are characterized by anisotropic properties inherent to crystalline solids (Cr) [1,2]. Specific properties of LCs are birefringence and dichroism, i.e., the different absorption of light rays propagating in different directions [3,4]. Due to the ability of LC molecules to change their orientation and, accordingly, optical properties under the action of weak short-term electric fields, they have found use in various industrial applications. Moreover, LCs have unique properties such as diamagnetism [5], memory effect [6], and low friction [7,8,9], which also increase their value.

There are three types of LCs according to the conditions of their transition to the mesomorphic state: lyotropic, metallotropic, and thermotropic. Lyotropic mesophases result from dissolving amphiphilic mesogens consisting of two parts with contrasting characters (e.g., hydrophilic and hydrophobic) in suitable solvents under appropriate concentrations and temperatures [10]. In this case, their properties depend not only on temperature but also on the nature and content of the solvent, which is generally volatile or/and hygroscopic, making the resultant mesophase unstable without barrier isolation. Metallotropic LCs consist of a phase including a surfactant and an organic solvent (or water) and an inorganic phase based on a soluble salt (e.g., ZnCl_2_), and their properties depend on the ratio of these phases besides the individual concentrations and temperature [11]. These mesomorphic systems have not found wide application, and the description of their properties is sketchy. Thermotropic LCs are usually single-component materials forming a mesophase state within a specific temperature range, which makes their behavior more predictable. This type of LC is more common, which is also due to the possibility of varying their properties by the structure of the mesogenic molecules consisting of flexible and rigid parts [12,13].

Thermotropic LCs include compounds that form the smectic (S), nematic (N), and cholesteric phases, which in turn can be calamitic, sanidic, discotic, or conic, depending on the shape of mesogenic molecules [14], and calamitic LCs are the easiest to synthesize and are hence the cheapest. Smectics are most similar to solids in structure because they tend to have tight packing [15], and calamitic smectogens consist of rod-like molecules that can arrange in parallel and form parallel layers [16]. The thickness of the smectic layers depends on the length of the mesogen molecules, and these layers move freely relative to each other, providing fluidity. The heating of a smectic phase may result in the transition to another smectic phase, a nematic phase, or an isotropic melt. In this case, the length of a flexible alkyl chain in mesogenic molecules has an essential influence on the properties of smectics, determining their type, thermal stability, and polymorphic behavior [17,18]. Calamitic nematic LCs also consist of rod-like unidirectional molecules but are less ordered than smectics since they do not form layers [19,20]. The structure of nematogens allows their molecules in the nematic state to slide easily relative to each other, making them highly fluid and able to change their molecular direction in dependence on external electromagnetic forces rapidly, which is essential for manufacturing liquid-crystal displays. Of all the LC types, nematics are most similar to liquids due to low viscosity and inelasticity and have a wide operational temperature range [21]. In turn, cholesterics take their shared name from cholesterol, and this type of LC mainly includes its different derivatives [22]. Cholesterics resemble smectics by layer structure, but every layer is monomolecular in thickness, and nematics by unified molecular orientation within every layer. They have a helical molecular structure: the direction of the long axes of the cholesteric molecules deflects by some angle in each successive layer. Since cholesterics consist only of chiral molecules, they have another name: chiral nematic LCs.

The type of generated mesophase depends on the molecular structure of the mesogen and, in turn, determines its physicochemical properties. A crucial characteristic of LCs is their viscosity, which decreases with an increase in temperature but then passes through a local maximum near the transition from a nematic phase to an isotropic melt and continues to decline monotonically [23]. This behavior appearing in a jump in viscosity is a consequence of the disruption of molecular organization with a transition from a unidirectional molecular order to a chaotic one. In this respect, nematic phases have a lower viscosity than the isotropic ones of the same molecules. However, smectic phases have a significantly higher viscosity than isotropic liquids as well as other LC types in comparable conditions [14,23,24,25,26]. Moreover, the viscosity of mesophases as anisotropic substances is not the same in different directions: along and perpendicular to molecular axes (in nematics) or layers (in smectics). Simple rheological tests at high shear rates estimate the viscosity close to the lowest one, i.e., to the longitudinal viscosity along molecular axes or layers of mesogens, whereas their transverse viscosity requires special techniques or a magnetic field in measuring [27,28]. Furthermore, depending on the temperature, one chemical compound can form various LCs having different viscosities.

Since mesomorphic systems of different types can be either fluidic or resistant to external actions, they are widespread in many biological and industrial processes. The structure and hence the viscosity properties of nematic LCs can be changed in a controllable way by chips and a low power of about several μW, which make them anisotropic and ensure the parallel arrangement of rod-like molecules relative to each other. Moreover, lyotropic high-molecular nematics allow for the production of high-strength polymer fibers, as the stretching of forming filaments enhances their strength because of the macromolecular orientation [29,30]. Cholesterics have found applications for creating memory cells capable of storing information for up to several years and as thermal indicators due to their high sensitivity to even the slightest changes in the ambient temperature [31]. Other applications of LCs are as charge-transfer matrices [32], lubricants [33,34,35,36], templating systems [37], superabsorbents [38,39], biosensors [40], electrical switches [41], pressure-sensitive adhesives [42], different shape-memory [43], stimuli-responsive [44], ion-conducting [45], and other functional materials [46], although the most common one is the creation of liquid-crystal displays for various devices [47,48].

Calamitic mesogenic molecules have a rod-like form, which is characterized by asymmetry due to the presence of rigid and flexible or polar and non-polar fragments, i.e., they have a complex structure produced by multistage synthesis, increasing their cost. However, mesomorphic systems can result from blending simple molecules containing carboxyl groups, which provide hydrogen bonds between these molecules with their nominal noncovalent dimerization into symmetric homodimers or asymmetric heterodimers. Usually, different *p*-alkylbenzoic [49] and *p*-alkyloxybenzoic [50,51] acids serve this purpose. Aromatic rings and carboxyl groups connected by hydrogen bonds represent the rigid part of rod-like dimers, while alkyl substituents at the rings are their flexible parts. For these acids, the length of the *n*-alkyl substituent must be at least three carbon atoms to make them mesogenic as pure compounds [52]. For example, *p*-methoxy- and *p*-ethoxybenzoic acids individually (in the form of homodimers) do not exhibit the LC state, but their heterodimeric mixture is in the mesomorphic (nematic) state, although in a small temperature region [53].

LCs based on mixtures of *p*-alkylbenzoic or/and *p*-alkyloxybenzoic acids are studied sporadically, usually considering no long alkyl substituents. Meanwhile, their blending could potentially lead to an increase in the temperature range of the existence of the liquid crystal state or even to obtain more highly ordered mesophases from simple molecules. Many short-substituted *p*-alkylbenzoic and *p*-alkyloxybenzoic acids alone do not exhibit liquid crystalline properties, while their binary blends can form a nematic phase [52]. However, *p*-*n*-octyloxybenzoic acid can be in the smectic state, while its mixtures with *p*-*n*-alkylbenzoic acids (*k*BA, *k* = 2, 5, 6, or 7) form only the nematic phase [54], meaning suppressing the organization of molecules into smectic layers. Nevertheless, mixtures of *p*-*n*-nonyloxybenzoic acid with *p*-alkylbenzoic ones can have several smectic phases [55], indicating the complexity of their phase behavior. Moreover, the rheological properties of these nemato- and smectogenic blends have not been investigated at all, despite their importance for practical applications. In the simplest case, the rheological properties can differ or not between the same mesophase types formed by identical molecules or by two different ones.

**Figure 1:**
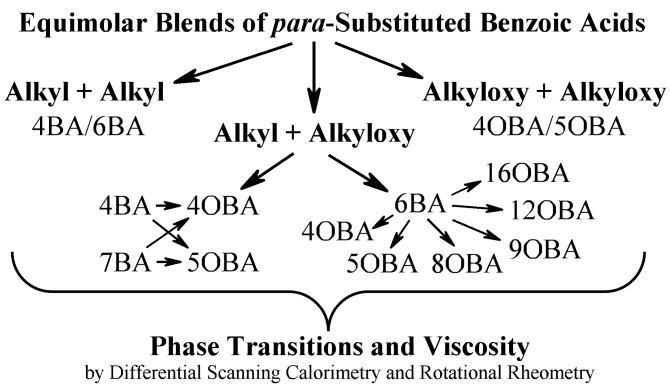
General research design: from simple to complicated combinations with their parallel comparison.

This work examines the phase transitions and rheological properties of LCs produced by blending *p*-*n*-alkylbenzoic and *p*-*n*-alkyloxybenzoic acids. We will first investigate a binary blend of two *p*-*n*-alkylbenzoic acids, then that of two *p*-*n*-alkyloxybenzoic acids, and finally proceed to their different combinations (Figure 1). In all cases, we will use equimolar blends of these acids to ensure that uniform heterodimers composed of two different acids can arise in the entire blend volume without homodimer impurities.

## 2. Results and Discussion

### 2.1. A Blend of Alkylbenzoic Acids (4BA/6BA)

**Figure 2:**
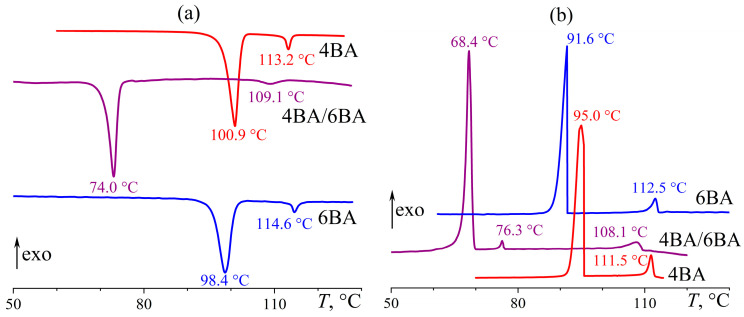
Heating (**a**) and cooling (**b**) thermograms of 4BA, 6BA, and their equimolar blends: an expansion of the mesophase state towards low temperatures and a new monotropic transition. Sample designations and peak temperatures are near the curves.

The individual acids 4BA and 6BA form the nematic phase in a narrow temperature range from 98–101 °C to 113–115 °C, expressed on the DSC curve as two endothermic transitions upon heating (Figure 2a). The conversion of the crystal phase into a nematic one (Cr–N) requires higher energy than the subsequent transition to isotropic liquid (N–I). The thermal transition from crystal to nematic state (rather than isotropic) is because the aromatic fragments of these acids retain their structuring due to weak π–π interactions. As a result, the crystal packing of mesogenic molecules divides into alternating loosely packed aliphatic and densely packed aromatic regions [56,57,58]. The dense packing of the aromatic ones is due to weak directional interactions such as CH…O and CH…N hydrogen bonds, π…π stacking, and CH…π bonds. In other words, the melting of a crystal occurs only in loosely packed aliphatic regions, whereas secondary bonds of the aromatic ones keep the system structured in a specific temperature range, forming an ordered melt—a mesophase [58,59,60]. Cooling causes reverse transitions of individual alkylbenzoic acids from the isotropic phase to the nematic one and then to the crystalline state (Figure 2b). In this case, the temperature of the N–I transition does not depend on the direction of temperature change, while crystallization occurs at a temperature about 3 °C lower than melting, i.e., the acids demonstrate the ability to weak undercooling (or supercooling [61]). Note that phase transition temperatures of low-molecular-weight substances are determined by the onset point of endothermic or exothermic transitions, unlike, e.g., polymers or multicomponent blends, whose transition points are usually measured by the peak location. However, it is not always possible to accurately determine the onset of transitions due to their blurriness for investigated acid blends. Therefore, the peak temperatures are shown in the DSC curves for comparison, but the onset temperatures of transitions were used for calculating undercooling correctly.

**Figure 3:**
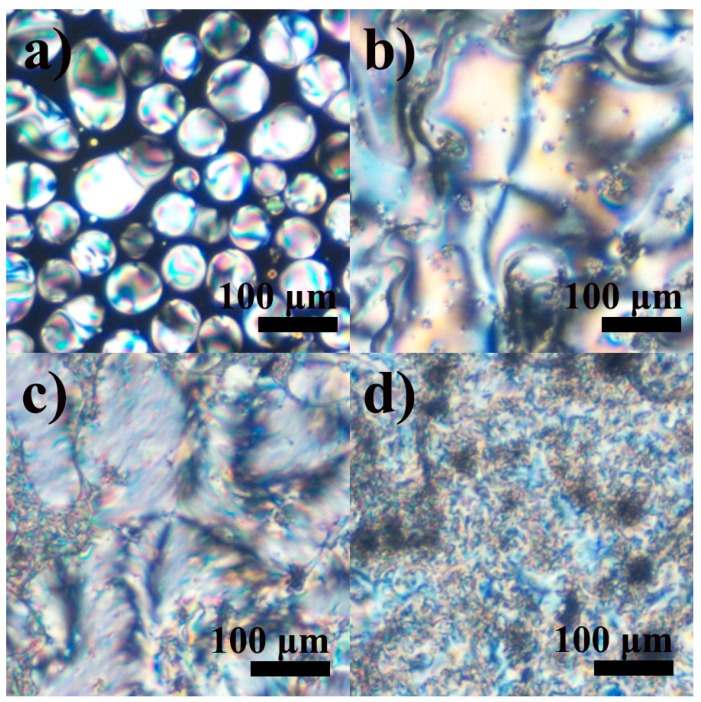
Microphotographs of the equimolar 4BA/6BA blend when cooled to 108 (**a**), 85 (**b**), 72 (**c**), and 36 (**d**) °C: nematic phase formation, nematic phase, new monotropic phase, and crystallization.

The blending of 4BA and 6BA significantly extends the region of existence of the LC state towards low temperatures by about 30 °C (Figure 2a). At the same time, the LC existence region is reduced by 4–5 °C at high temperatures. Moreover, a clear monotropic transition at 76 °C occurs upon cooling the blend without any pronounced undercooling during its subsequent crystallization (Figure 2b). In this case, the term monotropic means a liquid crystal to liquid-crystal transition that occurs below the melting point and is revealed by undercooling the liquid crystal, not the irreversible transition of a crystalline solid from a metastable polymorphic form to the stable polymorph [62,63]. Analysis of the blend’s microphotographs in crossed polarizers shows its nematic state in the temperature region of 76–108 °C: the formation of this phase occurs from round nucleates (Figure 3a) with the appearance of a schlieren texture (Figure 3b). Cooling to 76 °C causes the transformation of this smooth nematic texture into a rough brushed one with preservation of the initial positions of disclination lines (Figure 3c), which may be associated with the appearance of a smectic or a cybotactic nematic phase. Further cooling below 68 °C leads to crystallization with the formation of a rough and mottled texture (Figure 3d).

**Figure 4:**
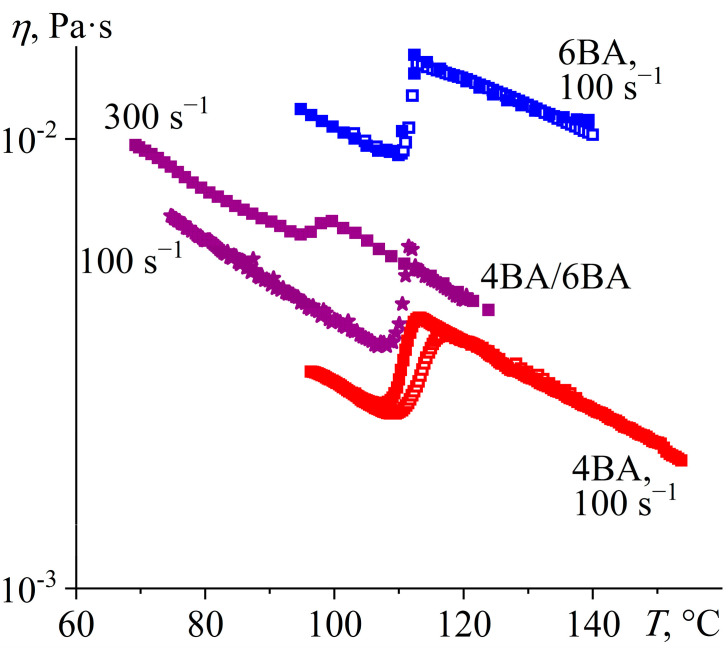
Temperature dependences of viscosity for 4BA, 6BA, and their equimolar blend in cooling (filled points) and heating (hollow points): the intermediate viscosity of the blend and the dependence of viscosity and N–I transition temperature on the shear rate due to the partial disruption of the nematic orientational order under extensive shear. Sample designations and shear rates are near the curves.

The individual acids 4BA and 6BA exhibit a clear transition to the nematic phase upon cooling their isotropic melt, which is manifested as a jump-like decrease in viscosity by about 40% (Figure 4). The lower viscosity of the nematic phase is due to the identical orientation of its molecules along the flow axis, whereas the molecules of the isotropic liquid are arranged chaotically even at high shear rates, accounting for its higher viscosity. Moreover, the viscosity of 6BA is almost four times higher than that of 4BA in both isotropic and nematic states, which is probably due to its higher molecular weight. The curves obtained by cooling the isotropic liquid and heating the crystal coincide, indicating the enantiotropic nature of these LCs. A slight divergence of the curves for 4BA in the region of the N–I transition is probably due to its tendency to slight undercooling and the delayed formation of an ordered structure. However, there is no such distinguishable undercooling in the form of viscosity hysteresis for 6BA, although DSC curves are comparable for 4BA and 6BA in terms of undercooling (Figure 2), implying the presence of an additional hidden factor. The analysis of these systems and those considered below showed that hysteresis is observed only for less viscous systems when the viscosity at the N–I transition is less than 5 mPa·s. Since the shear rate is the same (100 s^−1^), it means lower shear stress for less viscous systems: below 0.5 Pa. In addition, a comparison of the curves for systems with and without hysteresis reveals that the hysteresis is due to the shift of the curve obtained upon heating at the end of the nematic to the isotropic liquid transition as if the lower shear stress favors the stay of the system in the nematic state at higher temperatures. This conclusion is consistent with Figure 4, which shows that a higher shear rate and, correspondingly, higher shear stress causes a shift of the transition point for the 4BA/6BA blend towards lower temperatures. In other words, higher shear stress disrupts the orientational order of nematogenic molecules and makes their transition from the nematic to the isotropic state more abrupt, which is possibly due to secondary flows disturbing the laminar flow of the molecules [64,65].

The equimolar 4BA/6BA blend exhibits one N–I phase transition as a discontinuous viscosity change. The shear rate notably influences its temperature. At a shear rate of 100 s^−1^, the transition occurs at 108–112 °C, which coincides with DSC data (106–110 °C). An increase in the shear rate three times to 300 s^−1^ causes the transition temperature to decrease to 95–99 °C, which can be attributed to the influence of the applied mechanical field on the phase equilibrium. The intense shear induces secondary currents that destroy the nematic order of molecules, causing a shift of the transition temperature towards lower ones. In addition, the intensive shear with a rate of 300 s^−1^ disrupts the ordering of the nematic phase even after its formation, which causes an increase in its viscosity compared to that at 100 s^−1^. As a result, the viscosity jump due to the phase transition at 100 s^−1^ is more pronounced than at 300 s^−1^. In this case, the viscosity of the equimolar 4BA/6BA blend in both the isotropic and nematic states is closer in magnitude to that of the less viscous 4BA. At the same time, the blend’s monotropic transition has no appreciable effect on its viscosity, which is probably due to the high applied shear rates that disrupt the organization of molecules into the layered structure of a smectic or a cybotactic phase.

### 2.2. A Blend of Alkyloxybenzoic Acids (4OBA/5OBA)

**Figure 5:**
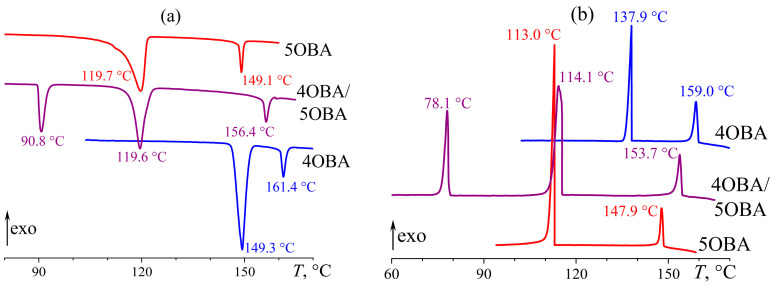
Heating (**a**) and cooling (**b**) thermograms of 4OBA, 5OBA, and their equimolar blend: an expansion of the mesophase state towards low temperatures and a new enantiotropic transition. The sample designations and peak temperatures are near the curves.

The individual alkyloxybenzoic acids 4OBA and 5OBA form nematic phases, like alkylbenzoic acids with comparable molecular lengths. However, Cr–N and N–I transitions occur at significantly higher temperatures (Figure 5). For the acid with a shorter butyl substituent, they happen at higher temperatures: the transition to the isotropic phase is observed at 149 °C and 161 °C for 5OBA and 4OBA, respectively, while their Cr–N transitions occur at 120 and 149 °C. Cooling of the individual acids induces the same transitions in the reverse order without appreciable undercooling.

**Figure 6:**
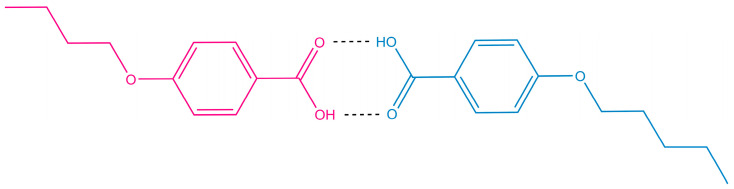
An example of an asymmetric dimer formed in an equimolar 4OBA/5OBA blend.

Blending 4OBA and 5OBA at an equimolar ratio leads to a new enantiotropic transition at 116 °C, whose position coincides with the crystallization point of 5OBA. In this case, this transition is not associated with the crystallization of this acid, as 5OBA is a less high-melting compound than 4OBA, i.e., the cooling of the blend 4OBA/5OBA could first crystallize 4OBA and then 5OBA, which is not observed experimentally. The blend’s N–I transition occurs at 155 °C, i.e., at an intermediate temperature compared to the N–I transitions of the individual acids. In turn, the melting of the blend happens at 90 °C, i.e., at a much lower temperature than 4OBA and 5OBA, implying the formation of asymmetric dimers 4OBA–5OBA (Figure 6) in the acids’ blend that crystallizes and melts as a whole as a result. However, the blend’s crystallization occurs at 79 °C with significant undercooling, indirectly indicating its high viscosity and the resulting retardation of the structural rearrangement.

**Figure 7:**
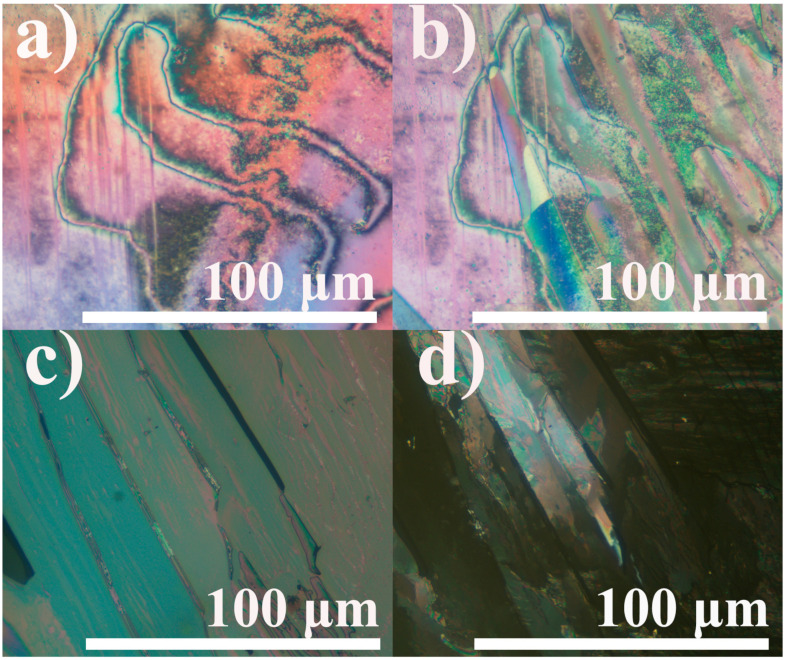
Microphotographs of the equimolar 4OBA/5OBA blend as it cools to 143 (**a**), 110 (**b**), 92 (**c**), and 68 (**d**) °C: nematic phase, enantiotropic transition, new enantiotropic phase, and crystallization.

Analysis of microphotographs of the equimolar blend 4OBA/5OBA in crossed polarizers shows the transition of isotropic liquid into the nematic phase with extended disclination lines (Figure 7a). Cooling to the temperature of the new transition at 116 °C induces parallel growth of the new phase with a mosaic smooth texture (Figure 7b). As this new phase cools, it grows, occupies the entire volume (Figure 7c), and then becomes dark and nontransparent due to crystallization (Figure 7d).

**Figure 8:**
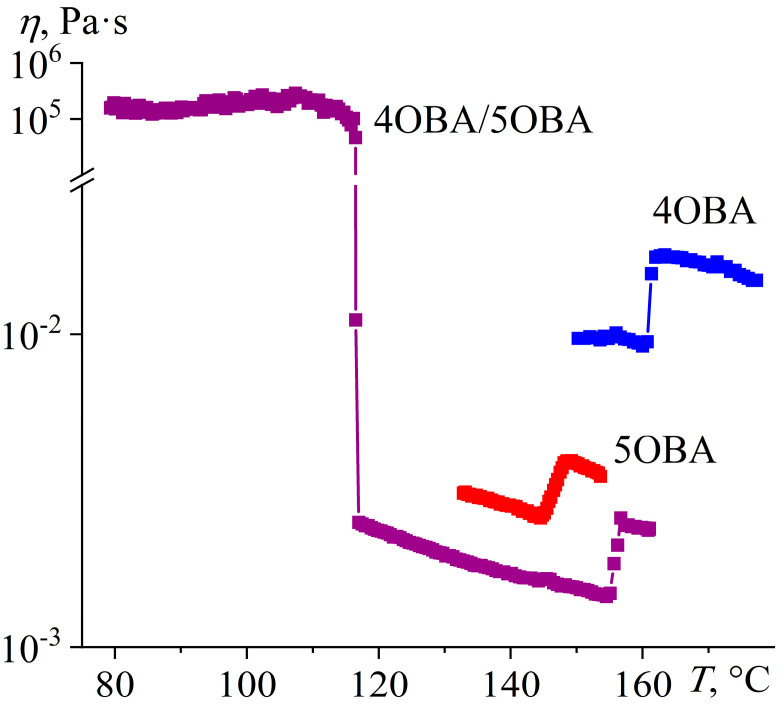
Temperature dependences of viscosity for 4OBA, 5OBA, and their equimolar blend: a very high viscosity of the new enantiotropic phase and a lower viscosity of the blend in the nematic and isotropic states. The designations of the samples are near the curves.

Measurement of the temperature dependences of the viscosities of the individual acids 4OBA and 5OBA shows that they exhibit a clear nematic–isotropic transition both at cooling and at heating since the viscosity of the ordered nematic phase is lower than of the chaotic isotropic phase (Figure 8). In this case, the viscosity of 4OBA is higher than that of 5OBA, which can be due to stronger intermolecular interactions (π–π bonds, H-bonds) in a molecule with a shorter aliphatic group. The higher strength of hydrogen bonds of 4OBA is also confirmed by its higher transition temperatures (Figure 5), i.e., the need for higher energy of molecular thermal motion to break intermolecular hydrogen bonds and disrupt the molecular order.

The mixing of two acids leads to a decrease in viscosity at high temperatures corresponding to the regions of nematic and isotropic states (Figure 8). Thus, the strength of intermolecular interactions is lower in the blend of these acids, which is also indirectly confirmed by the lower melting and crystallization temperatures than for the individual acids (Figure 5). However, the cooling of the blend below 117 °C increases its viscosity by seven decimal orders of magnitude while maintaining its ability to flow. In this case, the appearance of a smectic phase is probable, i.e., when the ordering of molecules into layers leads to the flow not of individual molecules but of their extended two-dimensional layers whose high viscosity is due to the numerous interlayer bonds between parallel layers, which must be broken to displace one layer relative to another. In other words, in the case of nematic or isotropic flow, the breaking of the intermolecular bonds of one molecule is necessary to move a molecule, whereas the breaking of the intermolecular bonds of the entire smectic layer is essential for smectic flow.

Thus, the blend of alkyloxybenzoic acids forms a new enantiotropic phase, whose high viscosity is characteristic of highly organized smectics. At the same time, the region of the LC state expands to low temperatures by about 30 °C, while the clearing point occupies an intermediate position between those of the original acids. The introduction of ether-group oxygen into the molecules of the acids’ blend leads to the transformation of the new phase from a low-viscosity monotropic to a high-viscosity enantiotropic one, a reduction in the viscosity of the isotropic and nematic phases, and a more substantial expansion of the liquid crystalline state with a higher decrease in the crystallization temperature due to a stronger undercooling. Let us consider how the transition to an asymmetric structure of mesogenic molecules due to combining alkylbenzoic and alkyloxybenzoic acids in their equimolar blend will affect the phase state and rheology.

### 2.3. Blends of Alkylbenzoic and Alkyloxybenzoic Acids

#### 2.3.1. Effect of Molecular Length Ratio (4BA/4OBA, 4BA/5OBA, 7BA/4OBA, and 7BA/5OBA)

**Figure 9:**
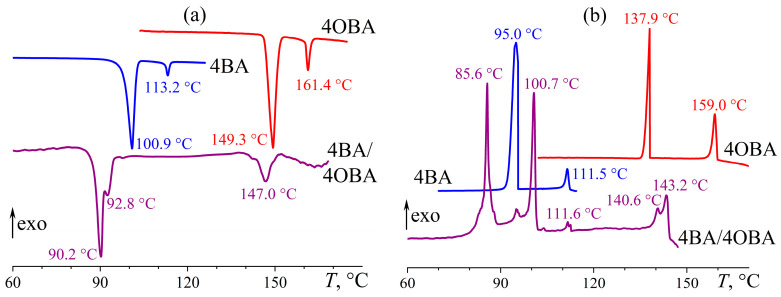
Heating (**a**) and cooling (**b**) thermograms for 4BA, 4OBA, and their equimolar blend: two-step transitions due to the formation of symmetric and asymmetric associates and new transitions upon cooling. Sample designations and peak temperatures are near the curves.

Equimolar mixing of alkylbenzoic and alkyloxybenzoic acids results in a blend with a complex thermophysical behavior characterized by several phase transitions (Figure 9). Upon heating, the blend exhibits a coupled transition from the crystalline to the nematic phase at 90–93 °C (Figure 9a). The coupled transition may be due to either a crystal-to-crystal transition (characteristic of some alkyloxybenzoic acids, e.g., 7OBA [66]) or a transition from the crystalline phase to the smectic phase and then to the nematic one. Further heating to about 147 °C causes the transition to the isotropic phase in a relatively wide temperature range (characteristic of blends of compounds), which may be due to the unequal structure of dimers 4BA/4OBA and the possible presence of impurities of homodimers 4BA/4BA and 4OBA/4OBA.

**Figure 10:**
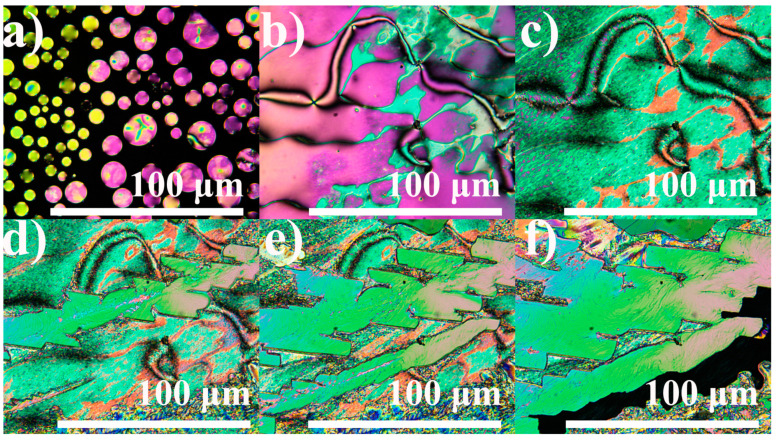
Microphotographs of the equimolar 4BA/4OBA blend when cooled to 143.0 (**a**), 120.1 (**b**), 104.4 (**c**), 98.0 (**d**), 93.2 (**e**), and 83.3 (**f**) °C: nematic phase formation, nematic phase, new monotropic phase, and multi-step crystallization.

**Figure 11:**
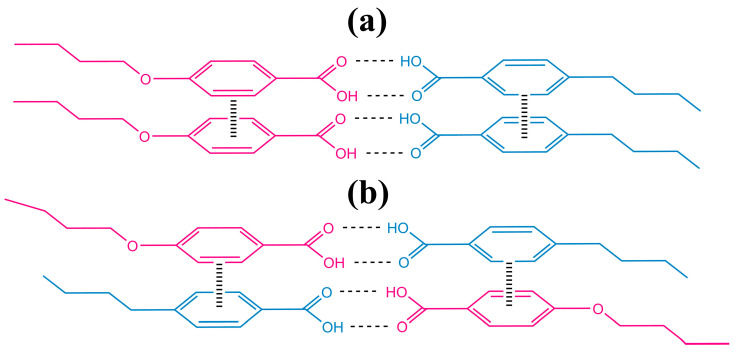
Formation of symmetric (**a**) and asymmetric (**b**) associates of alkylbenzoic and alkyloxybenzoic acids.

The cooling of the 4BA/4OBA blend leads to the formation of a nematic phase, judging by the typical spherical nucleation (Figure 10a). However, its appearance is accompanied by two exothermic peaks at 140.6 °C and 143.2 °C (Figure 9b), which may be associated with the formation of dimers and their associates having different structures. Dimer associates result from π–π interactions between the benzene rings [67,68]. However, the dimers in the equimolar blend are asymmetric: one side is from an alkylbenzoic acid, while the other represents an alkyloxybenzoic acid. As a result, dimer associates can be of two types: symmetrical and asymmetrical (Figure 11). In symmetrical associates, the aromatic rings of the acid that forms a part of the dimer interact with the aromatic rings of the same acid molecules. In asymmetrical ones, the aromatic rings of alkylbenzoic acids interact via π–π bonds with aromatic rings of alkyloxybenzoic acids and vice versa. It can be assumed that the symmetrical associates have a higher transition temperature than the asymmetrical ones, whose spatial structure can be disturbed by different lengths of hydrocarbon substituents of benzoic acids located parallel to each other, i.e., one under another in the associate. In addition, dipole–dipole interactions can also exist in symmetrical associates between the oxygen atoms of their ether groups, which may influence their transition temperatures and the type of mesophase formed.

Thus, the nematic phase of the 4BA/4OBA blend can consist of two types of associates. Meanwhile, it has a typical schlieren texture characterized by disclination lines (Figure 10b). Cooling to 111.6 °C induces a monotropic transition with a coupled heat release, indicating that both dimer associates undergo the transformation (Figure 9b). In this case, the texture changes from smooth to rough for the new monotropic phase (Figure 10c), which is similar to the monotropic nematic phase formation upon cooling the 4BA/6BA blend (Figure 3c). Further cooling to 100.7 °C causes an abrupt exothermic transition accompanied by the growth of the extended sawtooth-shaped crystals in the nematic phase (Figure 10d). There may be two explanations for the nature of these crystals. First, this transition may be due to the crystallization of 4OBA as the higher melting component of the blend. Second, there may be crystallization of one type of 4BA/4OBA dimer associates, which are probably the symmetric ones since higher transition temperatures would be expected for them due to the dipole–dipole interactions of their ether oxygen atoms and the symmetry of their associate structure (Figure 11a).

As cooling proceeds, the gradual growth of crystals happens (Figure 10e) until the remaining blend crystallizes at 85.6 °C with the formation of a nontransparent black texture (Figure 10f). In this case, two options are possible: crystallization of the remaining asymmetric associates or crystallization of pure 4BA. Since there is a double transition on the DSC curve when this blend is heated, and the transition temperatures are much lower than the melting points of the individual acids (Figure 9a), it can be concluded that the crystallization of associates rather than pure compounds occurs.

**Figure 12:**
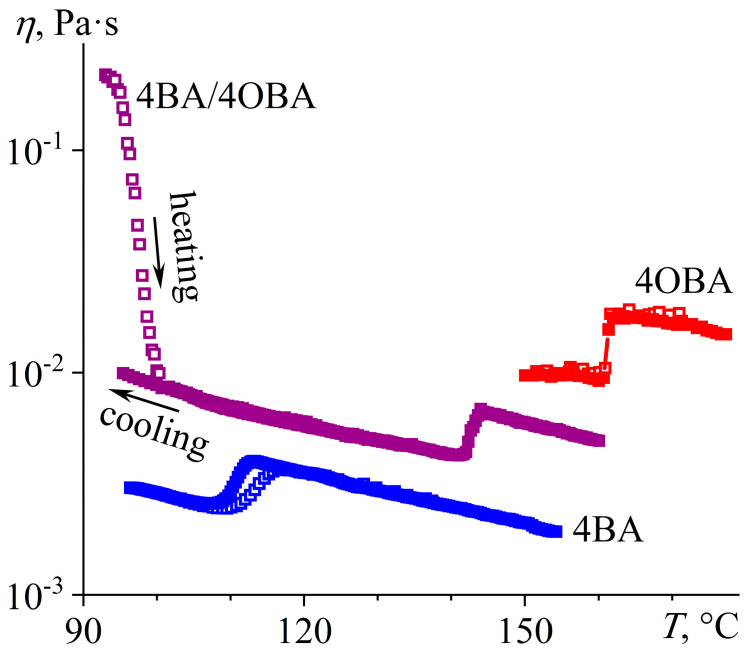
Temperature dependences of viscosity for 4BA, 4OBA, and their equimolar blend in cooling (filled symbols) and heating (hollow symbols): the intermediate viscosity of the blend, expansion of the nematic state, and appearance of low-temperature high-viscosity flowability due to the gradual melting of heterodimeric associates. Sample designations are near the curves.

In the isotropic and nematic states, the 4BA/4OBA blend has viscosities intermediate in value between those that are characteristic of the individual acids (Figure 12). At the same time, the transition from the crystalline state to the nematic one occurs in a relatively wide temperature range of 93–100 °C, which can be associated with a two-step Cr–N transition of first asymmetric and then symmetric associates (Figure 9a). The N–I transition should also occur through a two-step transition to the isotropic phase of asymmetric associates first (at a lower temperature) and then symmetric associates (at a higher temperature). However, according to rheometry data, the N–I transition is sharp and accompanied by an increase in viscosity at 142–144 °C, although the same N–I transition is extended in temperature with an endothermic peak at 147 °C according to calorimetry (Figure 9a). From the sharpness of the rheological transition and its lower temperature, we can conclude that the loss of nematic ordering by only one of the associate species (asymmetric in our case) disrupts the order of the symmetric associates and isotropizes the blend.

Meanwhile, the region of the nematic state in the 4BA/4OBA blend expands significantly, i.e., almost four times: from a temperature range equal to 10–12 °C for the individual acids to that of 40 °C for their equimolar blend. In this case, the expanding occurs due to a decrease in the crystallization temperature, which can be associated either with a lower crystallization temperature of 4BA/4OBA dimers or with suppression of their crystallization due to the presence of two types of associates that interfere with each other’s crystallization.

**Figure 13:**
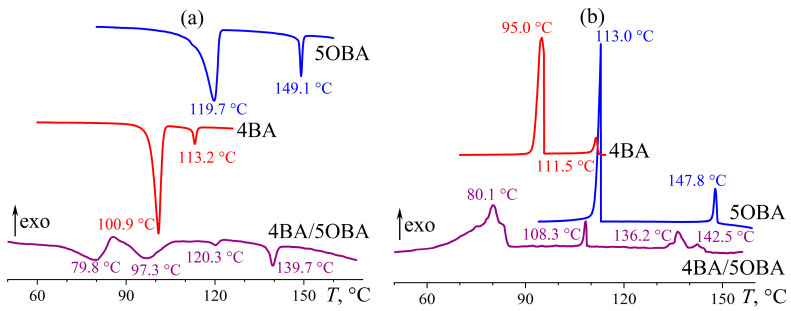
Heating (**a**) and cooling (**b**) thermograms of 4BA, 5OBA, and their equimolar blend: two-step gradual transitions of the blend and a new enantiotropic phase. Sample designations and peak temperatures are near the curves.

**Figure 14:**
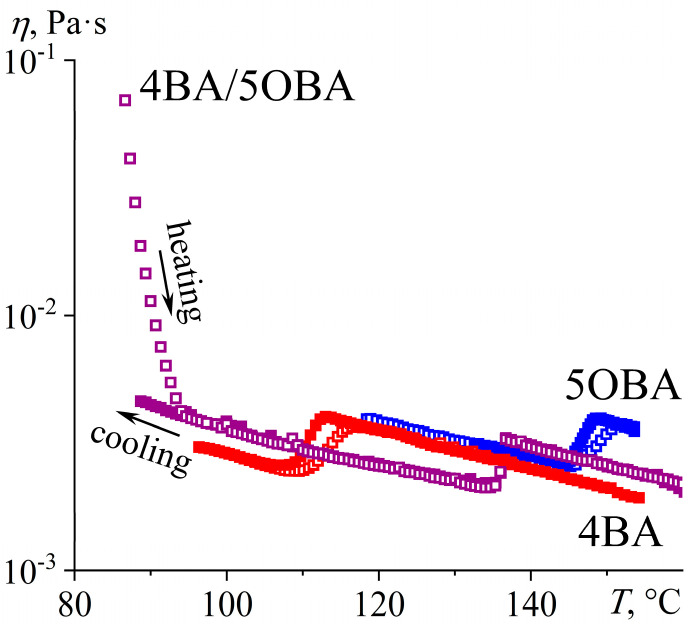
Temperature dependences of viscosity for 4BA, 5OBA, and their equimolar blend in cooling (filled symbols) and heating (hollow symbols): intermediate viscosity, expansion of the nematic state, and appearance of low-temperature high-viscosity flowability. Sample designations are near the curves.

The passage from the 4BA/4OBA to the 4BA/5OBA pair elongates the alkyl substituent in alkyloxybenzoic acid even more relative to alkylbenzoic acid. As a result, the asymmetry of non-symmetric associates increases, and they differ more strongly from their symmetric counterparts. The gain in the structural dissimilarity of the associates raises the difference between their transition temperatures (Figure 13). Upon heating the equimolar 4BA/5OBA blend, the melting of the asymmetric and symmetric associates occurs at 79.8 °C and 97.3 °C, respectively, and these transitions are blurred in temperature, possibly indicating a partial inhomogeneity in the structure of the dimers. The progressive melting of the associates leads to the fact that the viscosity of the blend decreases gradually, starting from the onset of fluidity at 87 °C (Figure 14).

At 120.3 °C, the 4BA/5OBA blend undergoes a transition according to the DSC data (Figure 13a), which, however, is not accompanied by a change in viscosity on the curve of its temperature dependence (Figure 14). Perhaps this transition is between different nematic states. At least the subsequent transition from the nematic phase to the isotropic one occurs at 139.7 °C with a characteristic jump-like increase in viscosity. In this case, the viscosity of the blend occupies an intermediate position between those of pure acids in both nematic and isotropic states.

The cooling of the blend from its isotropic state causes a two-step transition to the nematic state at 142.5 °C and 136.2 °C (Figure 13b), which can be associated with the formation of symmetric and then asymmetric associates from 4BA/5OBA dimers. At the same time, the viscosity jump occurs only at 136 °C, implying that the simultaneous formation of both types of associates (asymmetric and symmetric) is necessary for the actual transition to the nematic state. At 108.3 °C, a heat release occurs, which is probably the enantiotropic formation of a specific nematic phase that converts to the normal nematic phase at 120.3 °C. Cooling to 85–90 °C induces the onset of crystallization of the dimer associates, albeit widely extended on the temperature scale. Thus, similarly to other cases, the mix of two mesogenic acids extends the nematic state region toward lower temperatures.

**Figure 15:**
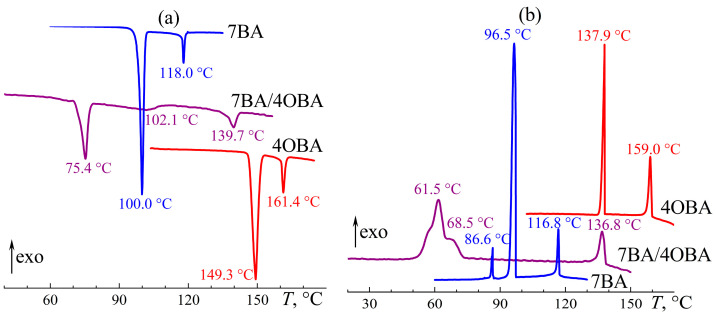
Heating (**a**) and cooling (**b**) thermograms for 7BA, 4OBA, and their equimolar blend: two-step gradual melting and crystallization and expansion of the nematic state. Sample designations and peak temperatures are near the curves.

At the passage from the 4BA/5OBA to the 7BA/4OBA blend, the absolute difference between the lengths of substituents at benzoic acids remains unchanged (two methylene groups), but then the length of the substituent of alkylbenzoic acid exceeds that of alkyloxybenzoic acid. This transformation leads to the suppression of the formation of symmetric associates, which is evident from the DSC curves (Figure 15). Upon heating the equimolar 7BA/4OBA blend, a pronounced endothermic transition occurs at 75.4 °C with the subsequent weakly pronounced one at 102.1 °C (Figure 15a). Since symmetric dimers presumably have higher transition temperatures, we can conclude that they are in the minority, possibly because of steric hindrance in their formation due to the increased length of the alkyl substituents compared to the above-discussed blends. At 139.7 °C, the isotropic liquid forms with the endothermic peak having a broad base, which is probably due to the different transition temperatures of the asymmetric and symmetric associates. The cooling of the isotropic blend causes a reverse transition to the nematic state at 136.8 °C (Figure 15b), followed by two-step crystallization at 68.5 °C (symmetric associates) and 61.5 °C (asymmetric ones).

**Figure 16:**
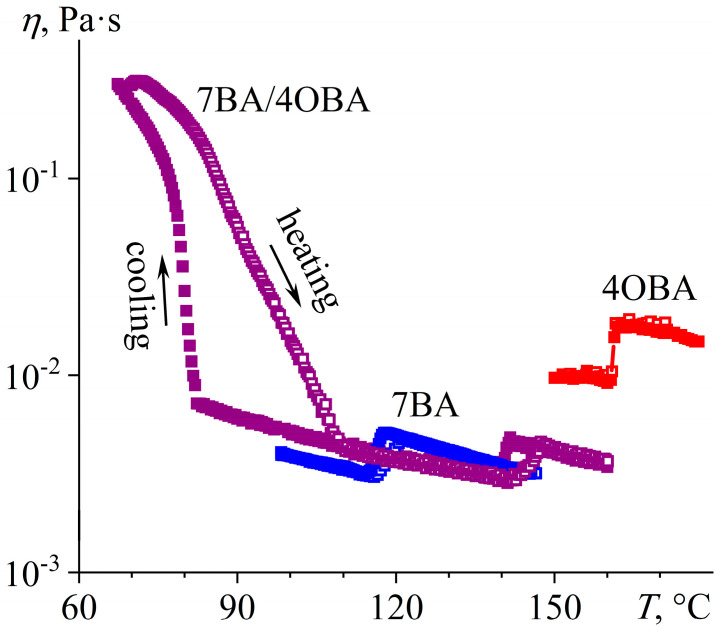
Temperature dependences of viscosity for 7BA, 4OBA, and their equimolar blend in cooling (filled symbols) and heating (hollow symbols): low-temperature high-viscosity flowability, gradual melting/crystallization of heterodimeric associates and their severe undercooling. Sample designations are near the curves.

The viscosity of the 7BA/4OBA blend is closer to that of the less viscous 7BA in both nematic and isotropic states (Figure 16). At the same time, the viscosity curves of the blend differ significantly in the low-temperature region with dependence on the direction of temperature change. Upon heating, the transition from the crystalline to nematic state is extended in temperature (67–109 °C), which can be due to the gradual melting of symmetric associates. Their gradual melting also results from the DSC data (Figure 15a): the peak at 102.1 °C has an extended shoulder that reaches the peak at 75.4 °C. Upon cooling, crystallization of symmetric associates occurs in a narrower temperature range, and the viscosity of the blend increases at a temperature changing from 83 °C to 67 °C with further loss of fluidity. Thus, symmetric associates are prone to significant undercooling, leading to the blend existing in a nematic state in a wide temperature range.

**Figure 17:**
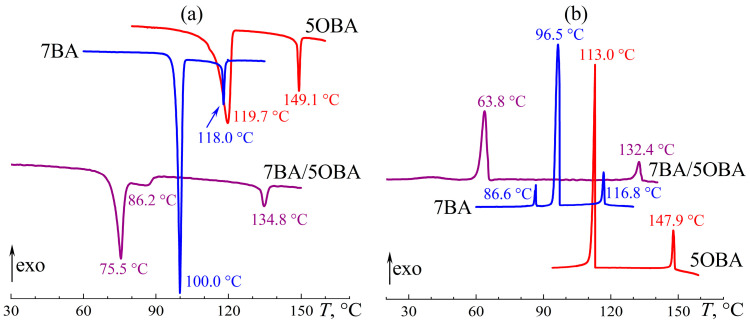
Heating (**a**) and cooling (**b**) thermograms of 7BA, 5OBA, and their equimolar blend: two-step melting of the blend and expansion of its nematic state. Sample designations and peak temperatures are near the curves.

A reduction in the length difference between the alkyl radicals of the acids to one methylene group in the 7BA/5OBA blend causes a decrease in the difference between the transition temperatures of the dimers’ associates. The melting of the asymmetric and symmetric associates occurs with an endothermic minimum at 75.5 °C and 86.2 °C, respectively (Figure 17a), whereas their crystallization is characterized by undercooling and happens at a single temperature of 63.8 °C with a uniform exothermic maximum (Figure 17b). The transition between nematic and isotropic states occurs also at a single temperature of about 133 °C. Thus, the N–I transition of the 7BA/5OBA blend has an intermediate temperature compared to the individual acids, while the region of the nematic state is significantly extended toward the low temperatures.

**Figure 18:**
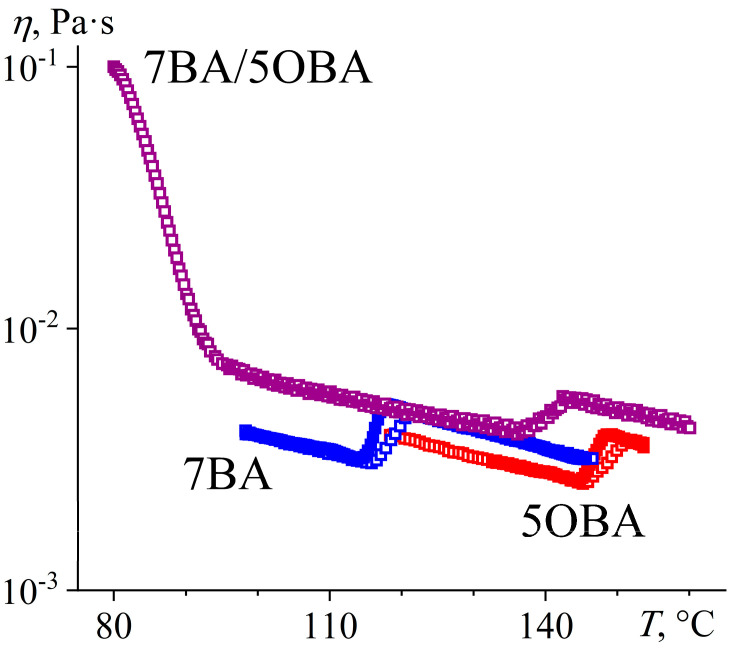
Temperature dependences of viscosity for 7BA, 5OBA, and their equimolar blend in cooling (filled symbols) and heating (hollow symbols): higher viscosity of the blend, expansion of its nematic state, and low-temperature flowability with high viscosity. Sample designations are near the curves.

When the 7BA/5OBA blend is heated from the crystalline state, its viscosity decreases smoothly in the temperature range of 80–95 °C (Figure 18), which can be due to the gradual transition of symmetric associates into the nematic state within these temperatures. Asymmetric associates transit into the nematic state at lower temperatures (with a maximum of 75.5 °C, see Figure 17a), but this transition does not cause melting of the blend as a whole with the appearance of fluidity. Thus, the transition of at least one form of associates from the crystalline phase to the nematic one is necessary for the realization of fluidity, whereas the transition of both forms of associates into the nematic phase is necessary for the formation of the nematic state by the blend as a whole. When heated to 137 °C, there is the start of a jump-like increase in the viscosity of the blend due to its transition from the nematic to the isotropic state, and this transition ends at 142 °C, i.e., it continues within the temperature range of 5 °C. For individual acids, the N–I evolution occurs within a narrower temperature range of 3 °C, which can result from the uniformity of the individual acids’ associates that are all symmetric. A blend of different acids contains symmetric and asymmetric associates and thus has a more extended temperature zone of the N–I transition.

Thus, the blends of alkyl- and alkyloxybenzoic acids form asymmetric heterodimers, which, in turn, form symmetric and asymmetric associates characterized by different transition temperatures. The difference in transition temperatures grows with the increased dissimilarity between the substituent’s lengths of the blended acids and the corresponding increase in the asymmetry of the asymmetric associates. To test this hypothesis, let us consider blends based on the same alkylbenzoic acid (6BA) to which we will add alkyloxybenzoic acids with increasing length of the hydrocarbon substituent: from 4OBA to 16OBA.

#### 2.3.2. Effect of Substituent Length (6BA/4OBA, 6BA/5OBA, 6BA/8OBA, 6BA/9OBA, 6BA/12OBA, and 6BA/16OBA)

**Figure 19:**
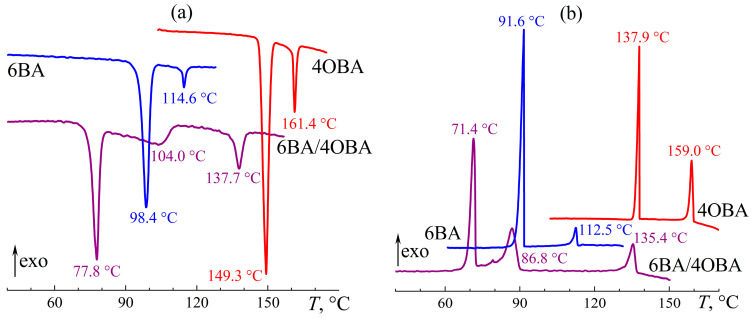
Heating (**a**) and cooling (**b**) thermograms of 6BA, 4OBA, and their equimolar blend: two-step gradual melting and crystallization of the blend and expansion of its nematic state. Sample designations and peak temperatures are near the curves.

In the 6BA/4OBA pair, the length of the 6BA molecules is longer by one methylene group. For this mixture, the transition associated with the crystallization or melting of asymmetric associates occurs sharply at a temperature of about 73 °C (Figure 19). Conversely, for symmetrical associates, the transition happens in a wide temperature range, especially in the case of their melting, which goes from the melting point of asymmetrical associates to the endothermic maximum at 104 °C (Figure 19a). During cooling, the mixture tends to undercool significantly, which results in the crystallization of symmetrical associates, occurring in a narrower temperature range than their melting (Figure 19b). The melting of the associates leads to the formation of a nematic phase, which transits to an isotropic phase in a single-step and reversible manner at 136 °C. As with other blends, the mix of 6BA and 4OBA leads to a twofold expansion of the temperature range of the nematic state compared to the individual acids.

**Figure 20:**
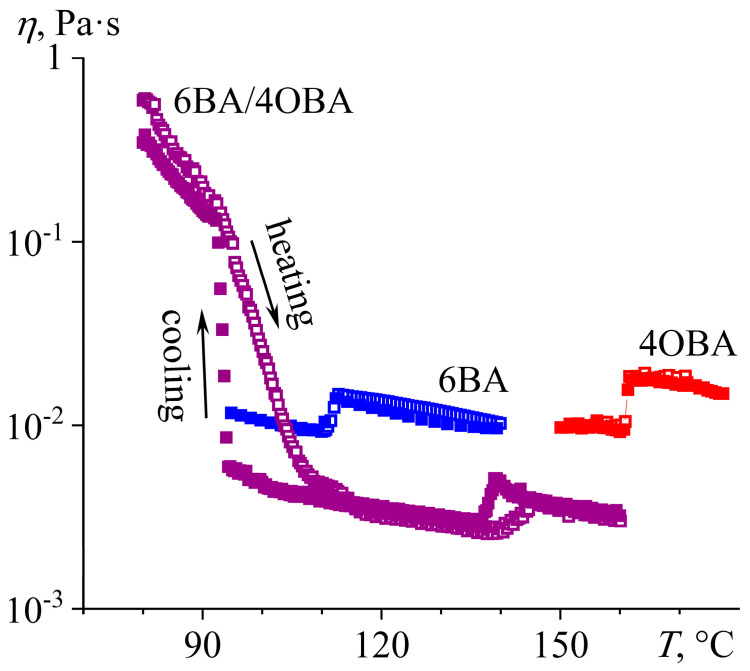
Temperature dependences of viscosity of 6BA, 4OBA, and their equimolar blend in cooling (filled symbols) and heating (hollow symbols): lower viscosity of the blend in the nematic and isotropic states, expansion of the nematic state, low-temperature high-viscosity flowability in a relatively broad temperature range, and gradual melting of symmetric associates with a gradual decrease in viscosity. Sample designations are near the curves.

The viscosity of the blend in the nematic and isotropic states is lower than that of the individual acids in the same forms (Figure 20). However, the blend’s N–I and Cr–N transitions are characterized by a noticeable viscosity hysteresis when comparing the viscosity curves in the modes of the increase and decrease of temperature. The viscosity hysteresis is most likely due to the difference in the transition temperatures of symmetric and asymmetric associates. The N–I transition for the acids’ blend occurs over a wider temperature region than for the individual acids, causing an increase in hysteresis. At low temperatures, the acids’ blend is prone to undercooling; because of this, the increase in viscosity due to the onset of crystallization of symmetric associates occurs at lower temperatures than the decrease in the viscosity because of their melting. The flow of the blend stops at a temperature of about 80 °C, i.e., when the asymmetric associates are in the crystalline state, unlike the symmetric ones. In other words, the flow is possible even when some associates are in the crystalline form (probably the symmetric ones, as they are hard to melt), but the flow will occur with high viscosity.

**Figure 21:**
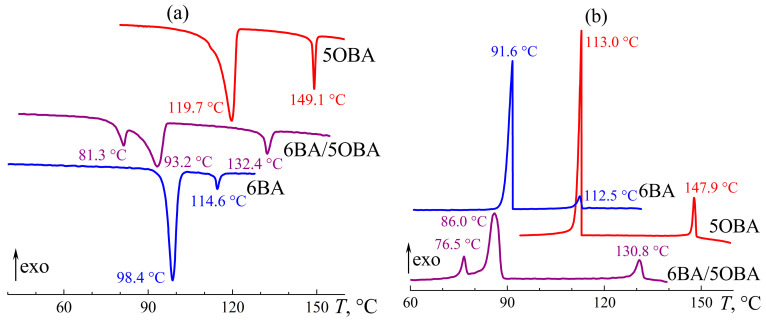
Heating (**a**) and cooling (**b**) thermograms of 6BA, 5OBA, and their equimolar blend: two-step melting and crystallization with a more pronounced high-temperature step and expansion of the nematic state. Sample designations and peak temperatures are near the curves.

The lengthening of the size of the substituent of alkyloxybenzoic acid by one methylene group leads to the 6BA/5OBA blend, where the acids’ molecules have practically the same length. The equal sizes of molecules mean the least distorted asymmetric associates when the acid molecules and their hydrocarbon substituents are strictly under each other in the dimer associates. This orthogonal structure makes these asymmetric associates related to the symmetric ones whose molecules are always arranged under each other for any pair of acids (see Figure 11). The transition from the crystalline to the nematic state and vice versa for this blend occurs in two steps (Figure 21), and the higher temperature one is more pronounced in energy. The latter may indicate a greater tendency of this blend to form symmetric associates, which is probably due to the similar lengths of these acids’ molecules. At higher temperatures, the N–I transition proceeds through a single stage, though being broader in temperature compared to the individual acids. Like other two-acid blends, the nematic state is temperature extended compared to the initial acids, and the extension occurs towards low temperatures.

**Figure 22:**
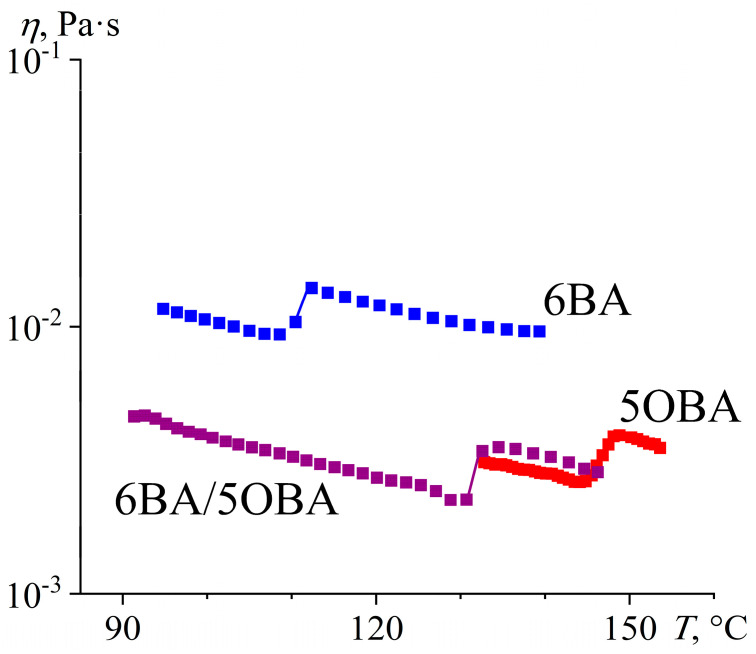
Temperature dependences of viscosity for 6BA, 5OBA, and their equimolar blend: lower viscosity of the blend and its non-fluidity at low temperatures that even partially crystallize heterodimeric associates. Sample designations are near the curves.

The viscosity of the blend is lower than that of the individual acids in both nematic and isotropic states (Figure 22). Upon cooling or heating, it immediately transits between nonfluid and low-viscosity nematic states, meaning that the fluidity requires the Cr–N transition of all associates, including the higher melting symmetric ones. Note that in other blends (6BA/4OBA, 7BA/5OBA, 7BA/4OBA, 4BA/5OBA, and 4BA/4OBA), the melting of the symmetric associates gave rise to flow with high viscosity. In these blends, the lengths of the alkylbenzoic and alkyloxybenzoic acids’ molecules differed, and the lower temperature Cr–N transition of the asymmetric associates was more pronounced or comparable in DSC curves in comparison with the transition of the higher melting symmetric associates. In other words, the formation of asymmetric associates is more typical for blends of differently long molecules, where fewer symmetric associates form, and their crystallization does not lead to flow stopping. In the case of the 6BA/5OBA blend, symmetric associates seem to prevail over asymmetric ones (at least the Cr–N transition of symmetric associates is more pronounced according to Figure 21), and, consequently, their crystallization makes the blend nonfluid in contrast to the other blends.

**Figure 23:**
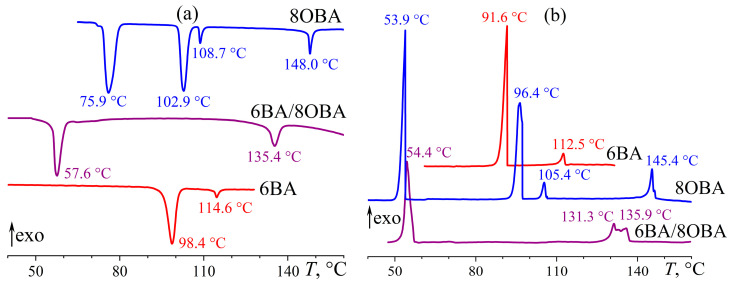
Heating (**a**) and cooling (**b**) thermograms for 6BA, 8OBA, and their equimolar blend: significant expansion of the nematic state and two-step I–N transition. Sample designations and peak temperatures are near the curves.

In the 6BA/8OBA blend, the length of the 8OBA molecule exceeds that of 6BA by three methylene groups. Moreover, 8OBA forms both the nematic (at 107–147 °C) and smectic (100–107 °C) phases and has a solid–solid transition at 74 °C (Figure 23). The mix of 8OBA and 6BA suppresses the formation of the smectic and second solid phases. The 6BA/8OBA blend transits from a crystal to a nematic state with an endothermic minimum of 57.6 °C and then to an isotropic liquid with a minimum of 135.4 °C. All transitions are single-step, so the presence of dissimilar associates with different transition temperatures is not revealed. The transition from the nematic to the crystal state also has a single-step pattern, in contrast to the transition from the isotropic liquid to the nematic phase. The latter probably forms through an intermediate phase, as the exothermic peak on the DSC curve has a dual shape at the I–N transition (Figure 23b). We can assume that the higher melting symmetric associates first form upon cooling and then transform into asymmetric associates (if symmetric and asymmetric associates coexisted in the blend, they would lead to a double transition during crystallization, just like in the other blends discussed above).

**Figure 24:**
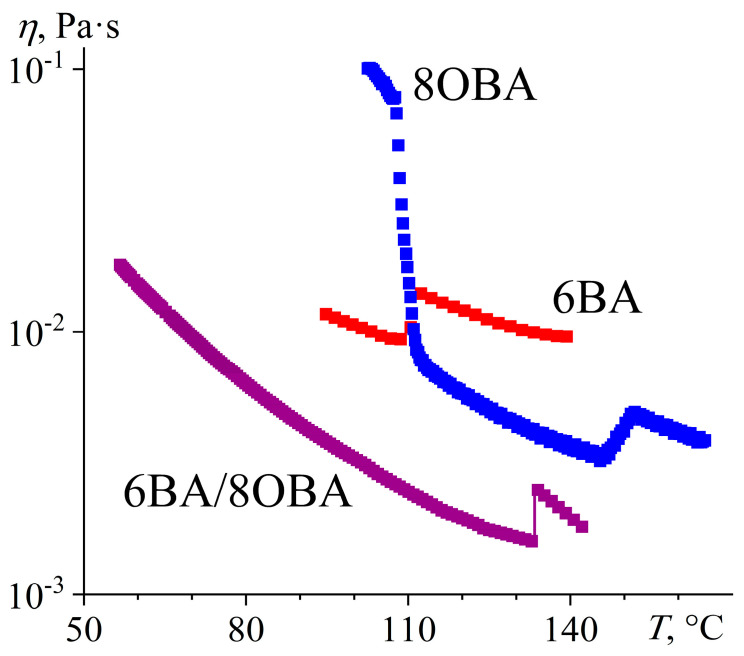
Temperature dependences of viscosity for 6BA, 8OBA, and their equimolar blend: lower viscosity of the blend, significant expansion of its nematic state, and suppression of smectic phase formation. Sample designations are near the curves.

The viscosity of 8OBA in the smectic state (at 100–107 °C) is one decimal order higher than its viscosity in the nematic state, and a jump-like drop in viscosity accompanies the transition between them (Figure 24). The smectic phase flows layer by layer, and every layer consists of numerous molecules, making the smectic viscosity higher than that of the nematic phase, whose molecules move individually. In turn, the viscosity of the nematic phase consisting of unidirectional molecules is lower than that of an isotropic liquid with chaotic molecular order. As a result, 8OBA changes to an isotropic liquid when heating with a characteristic viscosity jump in the temperature range of 146–151 °C. The mix of 8OBA and 6BA leads to a lower viscous blend and a significant expansion of the nematic state region to a range of 57–133 °C, i.e., to a width of 76 °C in absolute value compared to 14 °C and 33 °C for pure 6BA and 8OBA, respectively.

**Figure 25:**
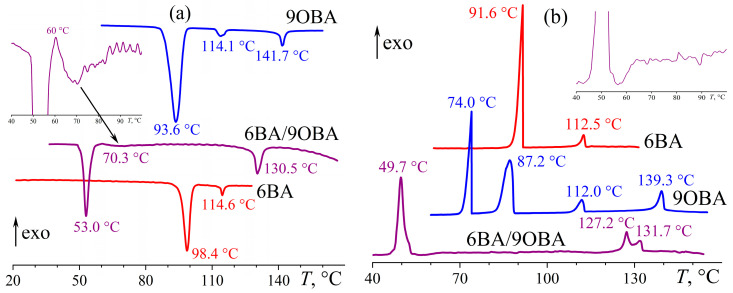
Heating (**a**) and cooling (**b**) thermograms for 6BA, 9OBA, and their equimolar blend: expansion of the blend’s nematic state, two-step I–N transition, and very low-energy enantiotropic transition at temperatures slightly higher than the melting or crystallizing ones. Sample designations and peak temperatures are near the curves. The insets show enlarged areas for the 6BA/9OBA blend in the region of its smectic–nematic transition.

Like 8OBA, 9OBA forms both smectic and nematic phases, but the temperature range of the smectic state is broader due to the shift of the melting towards lower temperatures (Figure 25). The mix of 6BA and 9OBA does not suppress the smectogenic nature of the latter: their equimolar blend can form both the smectic phase at low temperatures and the nematic one at high temperatures. In this case, the region of the nematic state expands to the temperature range of 60–130 °C, whereas that of the smectic state remains very narrow (like for initial 9OBA) but shifts substantially toward low temperatures (the S–N transition is observable only at a high magnification, Figure 25a). In addition, the initial 9OBA undergoes a monotropic solid–solid phase transformation at 75 °C, which is not evident for the 6BA/9OBA blend remaining in the nematic state at this temperature and crystallizing only at lower temperatures. Like in the case of the 6BA/8OBA blend, the formation of the nematic phase occurs in two steps with two heat release maxima, which can be due to the initial appearance of symmetric dimers and their subsequent transformation into asymmetric ones. At the same time, the N–S transition is indistinguishable from the baseline (inset to Figure 25b), which is possibly due to undercooling and superposition with the S–Cr transition; in any case, the DSC peak of the S–Cr transition has a low-temperature shoulder.

**Figure 26:**
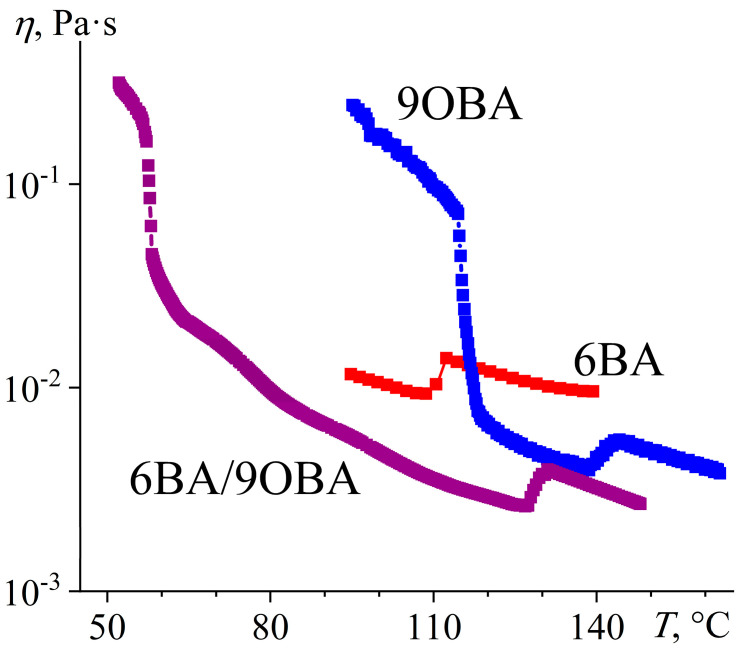
Temperature dependences of viscosity for 6BA, 9OBA, and their equimolar blend: lower viscosity of the blend in the nematic and isotropic states, significant expansion of the nematic state, and low-temperature appearance of the smectic phase. Sample designations are near the curves.

The temperature dependence of the viscosity of 6BA/9OBA shows the low-temperature region of a smectic phase, whose viscosity is comparable to that of 9OBA in the smectic state (Figure 26). A tenfold drop in viscosity accompanies the S–N transition, but it increases again but less intensively at the N–I transition. Thus, the realization of the smectic state is not obligatory but is possible in the case of blending nematogenic and smectogenic acids. In this case, the mix of 6BA and 9OBA makes the smectic phase more low-temperature, expanding the region of the nematic state and reducing the viscosity.

**Figure 27:**
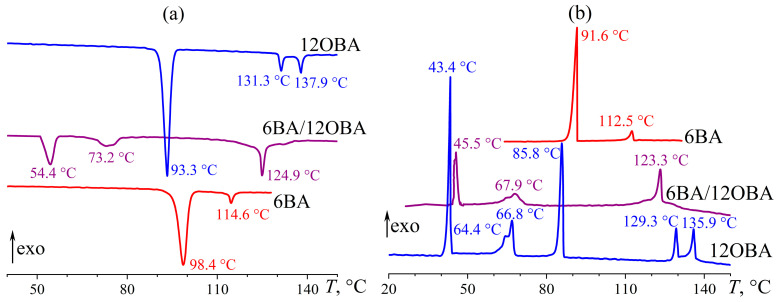
Heating (**a**) and cooling (**b**) thermograms of 6BA, 12OBA, and their equimolar blend: expansion of the mesophase state and broad transitions of the blend. Sample designations and peak temperatures are near the curves.

In contrast to 8OBA and 9OBA, 12OBA has a narrower nematic region (between 131 °C and 138 °C, Figure 27a) and a broader smectic one (between 93 °C and 131 °C). An equimolar combination of 12OBA and 6BA gives a blend having an expansive nematic zone and a relatively narrow smectic one with reduced transition temperatures (73–125 °C and 54–73 °C, respectively). The distinctly expressed two-step transition from isotropic liquid to nematic phase disappears, but the nematic-to-smectic evolution extends in temperature, which is perhaps due to the gradual transformation of dissimilar associates into uniform smectic layers crystallizing at a single temperature upon further cooling. Meanwhile, pure 12OBA exhibits three monotropic solid-phase transitions with exothermic maxima at 67 °C, 64 °C, and 43 °C (Figure 27b). The 6BA/12OBA blend also has N–S and S–Cr transitions at similar temperatures, most likely due to a coincidence.

**Figure 28:**
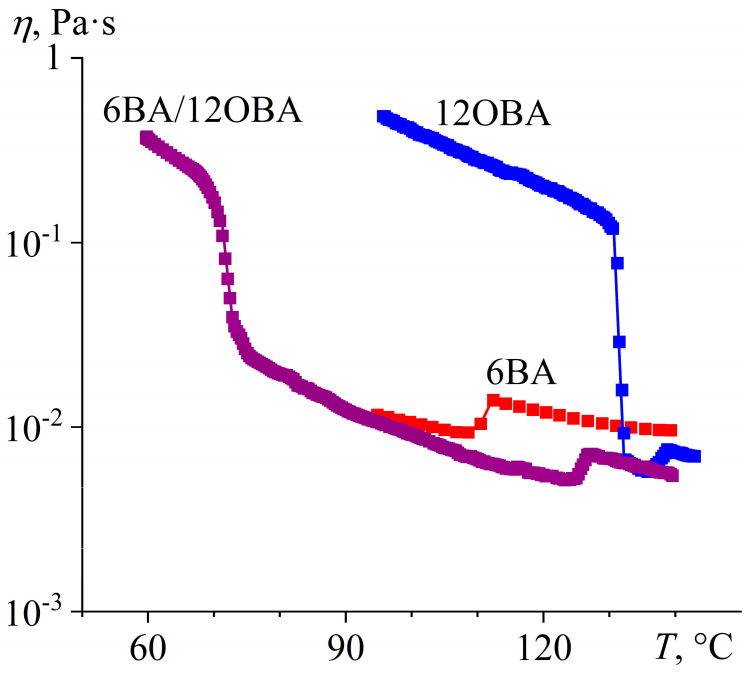
Temperature dependences of viscosity for 6BA, 12OBA, and their equimolar blend: lower viscosity of the blend in all states and expansion of the mesophase state towards low temperatures thanks to the broader temperature range of the nematic phase. Sample designations are near the curves.

The viscosity behavior of 6BA/12OBA is the same as that of 6BA/9OBA, except that the temperature region of the smectic state is wider (Figure 28). In addition, the viscosity of 6BA/12OBA is higher than that of 6BA/9OBA, which is probably due to the higher viscosity of 12OBA compared to 9OBA.

**Figure 29:**
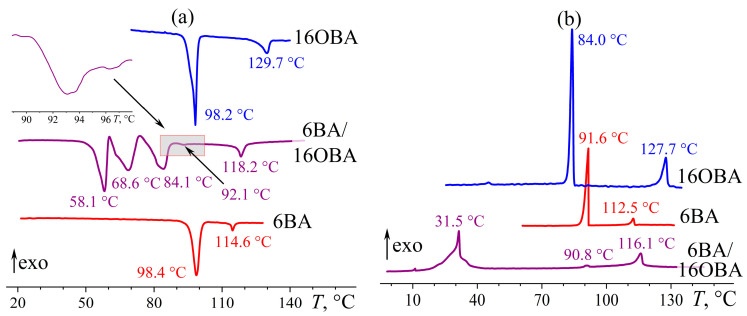
Heating (**a**) and cooling (**b**) thermograms for 6BA, 16OBA, and their equimolar blend: two solid–solid transitions before melting, a new slight enantiotropic transition, and significant undercooling. Sample designations and peak temperatures are near the curves. The inset shows an enlarged area for the 6BA/16OBA blend near its smectic–nematic transition.

**Figure 30:**
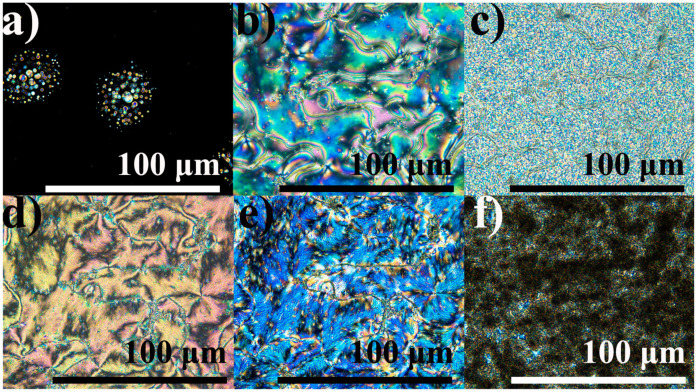
Microphotographs of the equimolar 6BA/16OBA blend when cooled to 116.7 (**a**), 108.0 (**b**), 89.1 (**c**), 86.0 (**d**), 49.9 (**e**), and 34.3 (**f**) °C: nematic phase formation, nematic phase, enantiotropic transition, smectic phase changing color, and crystallization.

The increase of the substituent length of alkyloxybenzoic acid up to hexadecyl radical leads to the absence of a nematic state in 16OBA: this compound forms smectic and crystalline phases at 128 °C and 100 °C, respectively (Figure 29). Nevertheless, the 6BA/16OBA blend forms a nematic phase from an isotropic liquid as there are typical round new-phase nuclei (Figure 30a) that merge into a schlieren texture (Figure 30b). At 89–91 °C, the blend undergoes a reversible transition accompanied by a relatively prolonged formation of the new phase through a “white noise”-like texture (Figure 30c) and almost indistinguishable peaks in the DSC heating and cooling thermograms (Figure 29). The white-noise texture then transforms into a new one that resembles the original nematic phase and retains the same disclination lines (Figure 30d). However, this new phase has a rough brushed texture in contrast to the silky glossy one of the initial nematic phase (Figure 30b). Upon cooling, the formed phase changes coloration without any change in texture (Figure 30e) and then transits to the crystalline state with the appearance of spherical dark regions (Figure 30f). In this case, crystallization occurs in a wide temperature range but has only one well-defined exothermic maximum at 31.5 °C (Figure 29b). The latter indicates that the crystallization happens with significant undercooling, as the 6BA/16OBA blend exhibits a phase transition upon heating only at 58.1 °C (Figure 29a), which, nevertheless, is a solid–solid one. Further heating leads to a second solid-phase transition at 68.6 °C and finally to melting at 84.1 °C. There are no solid-phase transitions in the cooling thermogram of this blend as it is in a liquid crystalline state at these temperatures due to undercooling.

**Figure 31:**
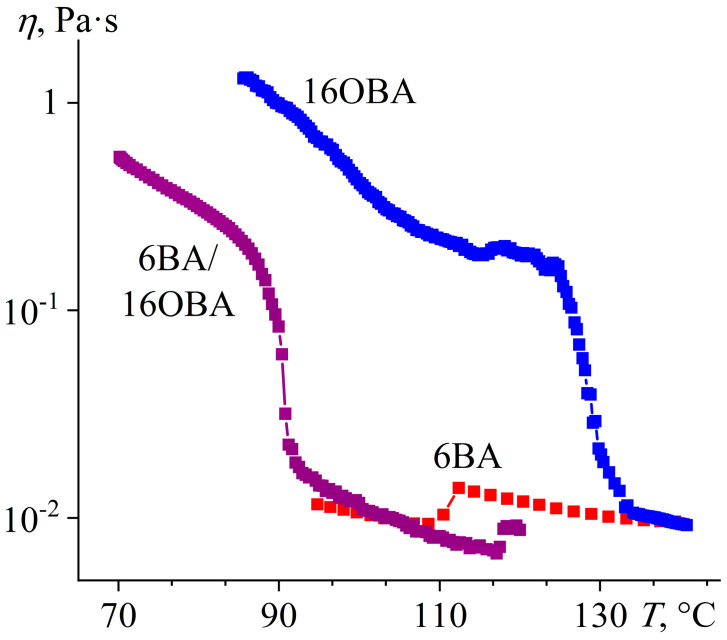
Temperature dependences of viscosity for 6BA, 16OBA, and their equimolar blend: formation of temperature-expanded nematic and lower-viscosity smectic phases at mixing purely nematogenic and smectogenic acids. Sample designations are near the curves.

According to viscometric data, the 6BA/16OBA blend forms a smectic phase since its viscosity at low temperatures below 90 °C exceeds that of the nematic and isotropic phases by a decimal order of magnitude (Figure 31). The viscosity of the 6BA/16OBA smectic is lower than that of the 16OBA one, which may be due to the reduced thickness of the smectic layers thanks to the shorter length of the asymmetric 6BA/16OBA dimers compared to the symmetric 16OBA/16OBA ones. Thus, the mix of smectogenic 16OBA and nematogenic 6BA leads to a slight expansion of the nematic region towards high temperatures and a shift of the smectic one towards low temperatures.

## 3. Materials and Methods

### 3.1. Materials

*p*-Alkylbenzoic acids (*k*BA, where *k* = 4, 6, and 7) and *p*-alkyloxybenzoic acids (*k*OBA, where *k* = 4, 5, 8, 9, 12, and 16) were supplied by Sigma-Aldrich (Darmstadt, Germany). Blended liquid crystals were obtained by mixing equimolar amounts of two alkylbenzoic or/and alkyloxybenzoic acids at temperatures above their melting points on a magnetic stirrer for 15 min. The following equimolar blends were obtained: 4BA/6BA, 4OBA/5OBA, 4OBA/4BA, 5OBA/4BA, 4OBA/6BA, 5OBA/6BA, 4OBA/7BA, 5OBA/7BA, 8OBA/6BA, 9OBA/6BA, 12OBA/6BA, and 16OBA/6BA. The choice of the equimolar composition of the blends is due to the desire to obtain only asymmetric dimers from two different acids without impurities of the initial symmetric dimers.

### 3.2. Methods

Transition temperatures of liquid crystals were measured by differential scanning calorimetry (DSC) on an MDSC 2920 analyzer (TA Instruments, New Castle, DE, USA) in the heating and cooling modes in an argon atmosphere at a temperature change rate of 2 °C/min.

Optical microphotographs in crossed polarizers were captured using an objective with 10× magnification and a 6PO microscope (Biomed Co., Moscow, Russia) equipped with an FP-82HT hot stage and an FP-90 controller (Mettler, Greifensee, Switzerland) at a cooling rate of 2 °C/min.

Rheological studies were performed on a Discovery HR-2 rotary rheometer (TA Instruments) using a cone/plate measuring unit with a plate of 25 mm diameter and an angle between the cone and plate of 2°. Temperature dependences of viscosity were measured at a constant shear rate of 100 s^−1^ and a temperature change rate of 2 °C/min. The rheological characteristics were calculated using the usual equations [69,70] with a relative error in their determination of no more than 5%.

## 4. Conclusions

The first-time systematic study of rheological properties and phase transitions in equimolar blends of mesogenic alkylbenzoic and alkyloxybenzoic acids revealed the following:

- The blending of alkylbenzoic acids or/and alkyloxybenzoic acids causes the formation of new asymmetric heterodimers that form a mesophase, just like these acids’ homodimers but with different transition temperatures and different viscosity properties.
- The blend of alkylbenzoic acids has a new monotropic transition to the second nematic phase, which is not accompanied by a change in viscosity, whereas that of alkyloxybenzoic acids has an enantiotropic transition to the new smectic phase having a very high viscosity.
- The blending of alkylbenzoic and alkyloxybenzoic acids probably leads to the simultaneous formation of symmetric and asymmetric associates (more and less high-melting, respectively) from their asymmetric dimers, at least the crystal–nematic and nematic–isotropic transitions in some cases occur in two-step manner according to the calorimetry data.
- High-viscosity flow of the *k*BA/*k*OBA blends at the crystal–nematic transition is possible, even if a part of symmetric associates is in the crystalline state; however, for the realization of the low-viscous nematic state, the metamorphosis of both forms of associates into the nematic phase is necessary.
- For the transition of an isotropic liquid to the nematic state, the formation of asymmetric associates is necessary (as formed at lower temperatures), whereas their loss of nematic ordering destroys the symmetric associates’ order and makes the blend an isotropic liquid.
- The blending of alkylbenzoic and alkyloxybenzoic acids with short radicals (butyl- or pentyl-) leads to a new mono- or enantiotropic transition to the second nematic phase without changing the viscosity at this evolution.
- The blend of alkylbenzoic and alkyloxybenzoic acids with the same molecular lengths has distinct transitions of symmetric and asymmetric associates between their crystalline and nematic states. A decrease in the alkyloxybenzoic acid’s molecular size leads to a broadening of the melting region of the symmetric associates, while its increase causes a two-step or even blurred isotropic–nematic transition, which is possibly due to the transformation of initially formed symmetric associates into the asymmetric ones.
- The blending of smectogenic and nematogenic acids can suppress the formation of the smectic phase if its temperature range for smectogenic molecules is low, whereas the nematic state forms even if one of the acids is not a nematogen.
- The blending of *p*-*n*-hexylbenzoic and *p*-*n*-octyloxybenzoic acids forms a mesogen having the most extended temperature range for a nematic state, including low temperatures (from 57 °C to 133 °C, 76 °C in difference). In turn, the mixing of *p*-*n*-butyloxybenzoic acid and *p*-*n*-pentyloxybenzoic acid gives the highest-temperature nematic phase with the broadest existence region (up to 156 °C), while the combination of *p*-*n*-hexylbenzoic acid and *p*-*n*-nonyloxy- or *p*-*n*-dodecyloxybenzoic acid results in the lowest temperature smectic phase (down to 51 °C).

The main advantage of this work is the demonstration that blending of easily accessible mesogenic compounds allows for extending the temperature range of existence of the liquid crystalline state toward low temperatures and reducing the viscosity of the resulting mesophase, i.e., accelerating its response to external actions. Its disadvantages are the non-examination of non-equimolar mixtures for the construction of phase diagrams and the hypothetical assumption of the existence of symmetric and asymmetric associates of heterodimers of alkyl- and alkyloxybenzoic acids, which should be investigated in more detail. Thus, further research works should aim at investigating the structure of equimolar blends of alkyl- and alkyloxybenzoic acids, modeling the formation of their symmetric and asymmetric associates to specify the causes of viscosity changes upon acids’ blending, and extending the work to non-equimolar blends and blends of other mesogenic compounds forming H-bond dimers.

## Data Availability

The data presented in this study are available upon request from the corresponding author.
